# Usefulness of the middle cerebellar peduncle approach for microsurgical resection of lateral pontine arteriovenous malformation

**DOI:** 10.3171/2020.10.FOCVID2063

**Published:** 2021-01-01

**Authors:** Shinsuke Tominaga, Miyahito Kugai, Kou Matsuda, Keisho Yamazato, Toshihiko Inui, Masahiko Kitano, Hiroshi Hasegawa

**Affiliations:** Department of Neurosurgery, Tominaga Hospital, Naniwa-ku, Osaka, Japan

**Keywords:** lateral pontine arteriovenous malformation, surgical treatment, middle cerebellar peduncle approach, pial transection technique, video

## Abstract

Surgical treatment of brainstem arteriovenous malformation (AVM) is challenging and associated with a higher risk of complications and a lower rate of gross-total resection. The authors present their experience with the surgical management of lateral pontine AVM using the middle cerebellar peduncle approach. All cases presented with neurological deficits that were caused by hemorrhage before surgery. In all cases, the AVM was not visualized on postoperative angiography, and there was no deterioration of neurological symptoms. In this video, the authors report the treatment results of one case and describe the technique with a review of the literature.

The video can be found here: https://youtu.be/bFvEMtMnrKw

## Transcript

**0:33** Surgical removal of the lateral pontine arteriovenous malformation (AVM). Lateral pontine AVM was located outside the trigeminal root within the cerebellopontine angle (CPA). The upper, lower, and lateral surfaces of the nidus were in contact with noneloquent cerebellar areas. From historical experience, when an approach through the middle cerebellar peduncle is considered a safe entry zone, it is utilized to arrive at the pontine tegmentum from the apparently noneloquent lateral pontine surface. Even if the pontine tegmentum is reached, postsurgical morbidity due to manipulation injury during the surgery is unlikely. The pial and parenchymal types of lateral pontine AVM are considered to have safe resectability. Arterial supply is mainly through the anterior inferior cerebellar artery (AICA). The superior cerebellar artery is a supplementary feeder, making it easy to adapt for removal due to the involvement of the superior petrosal vein as a main drainage.^1–5^

**1:37 Surgical Approaches to Lateral Pontine AVM.** Illustration demonstrating three surgical corridors to lateral pontine arteriovenous malformation.^3,6^

**1:57 Case Presentation.** In the last 3 years, 5 cases of the lateral pontine AVM were operated via lateral suboccipital retrosigmoid approach in our institution. In all cases, angiographically complete removal were performed and postoperative neurological status of the patients were unchanged or even improved. Here, we present one of the representative cases. The case is a 30-year-old male with a history of two bleeds and confirmed hearing impairment. The AVM was located from the right cerebellopontine angle to the right middle cerebellar peduncle and to the lateral pontine tegmentum. Lateral and medial branches of the right AICA were dominant feeders. Drainage was provided by two petrosal veins. The petrosal vein coming from the vein of the cerebellopontine fissure was the main drainer, and the petrosal vein coming from the vein of the middle cerebellar peduncle was assistant drainer. The nidus was irregular shaped, 24 mm long, mostly compact, and partly diffuse. It was classified as grade 2 on Spetzler and Martin grading scale.

**2:51 Strategy.** The right lateral suboccipital retrosigmoid approach was selected and splitting of the petrosal fissure was planned. Extent of the nidus was from the epipial layer to the pial layer, and the remaining section was of mixed type, extending through the middle cerebellar peduncle to the lateral pontine tegmentum with a parenchymal component. Since the lateral pontine surface is traditionally noneloquent and the middle cerebellar peduncle is considered a safe entry zone to enter the brainstem, intrusion into the brain parenchyma of the pons may be permissible to some extent. Pial and parenchymal dissection was conducted based on the guidance of various monitoring systems. At times, the pial components were removed using pial transection technique. It should be noted that some of the parenchymal components have to be left in a lifeless state using pial transection technique.

**3:45 Pial Transection Technique.** Velat et al. described the pial transection technique in removing perimedullary spinal AVMs.^7^ In a mixed AVM, when a portion of the nidus is on the brain surface and the remainder is in the brain, the pial surface of the brainstem or all the upper pial components, feeders, and drainers are occluded initially. The nidus is separated from the parenchymal component and only the pial component and the main drainer are removed. This technique is helpful for brainstem and spinal AVMs but should not be considered appropriate for most supratentorial brain AVMs sitting within the brain parenchyma. The pial transection technique requires a plane or “line in the sand” where violation of a certain eloquent region, such as the brainstem or spinal cord, is avoided.

**4:34 Presurgical Embolization.** The rostrolateral and caudomedial branches arose separately from the AICA. Rostrolateral branch was a feeder and two lateral and medial branches arising from the rostrolateral branch were occluded. In the postembolization images, a decrease in the size of the nidus and flow to the nidus were confirmed. These findings suggested that dearterialization of the nidus had progressed.

**4:58** The petrous fissure was separated, followed by opening of the cerebellomedulary cistern and the cerebellopontine cistern. During this procedure, the vein of the cerebellopontine fissure, which separated the superior semilunar lobule and the middle cerebellar peduncle, was exposed. A large tense varix appeared deep in the central section of the supratrigeminal triangle. Indocyanine green angiography was performed during the surgery. We confirmed that sufficient dearterialization had been achieved.

**5:32** An attempt was made to occlude the main feeders. Initially, the AICA medial branch from the supratrigeminal triangle was occluded with a clip. Subsequently, we coagulated and clipped the AICA lateral branch leaving the CPA cistern through the infratrigeminal triangle. The trigeminal nerve and the petrosal vein were retracted upward to open the infratrigeminal triangle. Numerous feeders, which ran across the brain surface toward the anterior lateral pontine base through the infratrigeminal triangle, were coagulated and cut.

**6:21** Approaching the supratrigeminal triangle again, a feeder branching from the AICA was found and occluded with a clip. When the lateral pontine surface was peeled off, a xanthochromic area was found directly below the surface.

**6:53** The main drainer was retracted upward, extensively revealing the anterior pontine surface. A drainer was located behind the supratrigeminal triangle, which prevented the operation. It was thought that removal of this drainer would facilitate a smooth surgery. To determine whether this drainer could be cut at this stage without complications, we traced it to the point where it entered the superior petrosal vein. Test occlusion was conducted on this drainer. No bleeding was noted during the 5-minute period of occlusion. No color change or swelling of the nidus or the drainer was observed. The drainer was occluded and cut.

**7:42** Subsequently, we moved on to the treatment of the nidus located in the middle cerebellar peduncle. Except the areas that could be coagulated and cut, bleeding sections within the brain parenchyma were treated only by pial transection technique.

**8:58** Removal of the nidus of the superior lateral pontine surface allowed wide opening of the supratrigeminal triangle. The outer edge of the anterior pontine surface was exposed. The diffuse nidus surrounding the trigeminal nerve root exit zone was coagulated and removed. Nidus was buried within the xanthochromic brain parenchyma directly below the lateral pontine surface. This section was treated by pial transection technique, since removal of this section of the nidus would have inevitably damaged the brain parenchyma.

**9:43** The supratrigeminal triangle and the deeper anterior pontine surface could be visualized. No abnormal blood vessels were found. Intraoperative angiography was performed to confirm the lack of residual arteriovenous shunting.

**9:59** No lesions were visualized on postoperative angiography. The patient experienced no postoperative numbness from heat or manipulation to the trigeminal nerve.

**10:09 Conclusions.** The middle cerebellar peduncle approach is extremely important for minimizing postoperative neurological deficits.

## Author Contributions

Primary surgeon: Tominaga. Assistant surgeon: Kugai, Matsuda. Editing and drafting the video and abstract: Hasegawa. Critically revising the work: Kitano. Reviewed submitted version of the work: Yamazato, Inui, Hasegawa.
